# A qualitative study exploring the health-related quality of life and symptomatic experiences of adults and adolescents with ulcerative colitis

**DOI:** 10.1186/s41687-019-0154-x

**Published:** 2019-10-30

**Authors:** Louise Newton, Jason A. Randall, Theresa Hunter, Shannon Keith, Tara Symonds, Roberta J. Secrest, Wendy J. Komocsar, Sarah E. Curtis, Linda Abetz-Webb, Michael Kappelman, April N. Naegeli

**Affiliations:** 1 Clinical Outcomes Solutions, Unit 68 Basepoint, Shearway Business Park, Shearway Road, Folkestone, Kent, CT19 4RH UK; 2 Eli Lilly and Company, Indianapolis, Indiana, USA; 3 Clinical Outcomes Solutions, Chicago, USA; 4 Patient-Centered Outcomes Assessment, Stockport, UK; 5 University of North Carolina, Chapel Hill, USA

**Keywords:** Ulcerative colitis, Adult, Adolescent, Qualitative, Experience

## Abstract

**Background:**

Ulcerative colitis (UC) often first presents during adolescence and early adulthood. Primary symptoms of UC are well known, yet similarities and differences of disease experience in adults and adolescents are not well characterized.

**Methods:**

To understand the health-related quality of life (HRQoL) and symptomatic experience of UC, in-depth interviews were conducted in the US with 21 adults (20–70 years) and 14 adolescents (12–17 years). Eligibility and medical history were confirmed by clinician report. A previously conducted literature review and resultant conceptual model informed the discussion guide to explore symptoms and HRQoL. Age appropriate creative tasks (“animal” task and collage) were employed to facilitate discussion. Transcripts and collages were subjected to thematic analysis using ATLAS.ti software.

**Results:**

Clinician-reported UC severity included 24% mild, 38% moderate, 38% severe among adults; and 64% mild, 29% moderate, 7% severe among adolescents. Among adults, 52% were female, 67% were white. Among adolescents, 50% were female, 71% were white. During analysis it was noted that all participants reported stomach/abdominal pain. Other key symptoms identified were frequent bowel movements, diarrhea, blood in stools, sudden need for bowel movement, stomach cramping, bloating, and feeling gassy/passing gas (≥75% of participants). Key impacts identified were embarrassment, dietary limitations, having to plan around UC, worry/fear, anger, low mood/depression, and relationship with others, (≥75% of participants). In creative tasks, animals were chosen to represent their UC and content included in the collages reflected the most commonly discussed themes from the interviews. Only adults discussed feeling dehydrated, while only adolescents discussed the impact of UC on school life.

**Conclusions:**

Open-ended interviews highlighted the HRQoL and symptomatic experiences of UC from the patient’s perspective, which were similar between adult and adolescent UC patients.

## Background

Ulcerative colitis (UC) is a chronic gastrointestinal disease, which often presents in adolescence and early adulthood, and is characterized by inflammation in the colon [1]. The primary symptoms of UC are well known and include blood in the stool, diarrhea, and abdominal pain, all of which can reduce overall health-related quality of life (HRQoL) [2, 3]. While the primary symptoms of UC are well-recognized, other aspects of the HRQoL and symptomatic experience of adults and adolescents have not been well-documented, particularly in the younger population. Moreover, scientific examination of the differences and similarities in the symptoms and impacts experienced by adolescents and adults with UC is limited.

Patients diagnosed with UC in childhood typically have more extensive disease, with more frequent, acute, and severe exacerbations when compared to those diagnosed as adults [2, 3]. Regardless of age, those with UC report intermittent disease flares interspersed with periods of remission, with approximately 20–30% requiring colectomy within 10 years of diagnosis [1].

The United States (US) Food and Drug Administration’s 2009 guidance on the use of patient-reported outcome (PRO) measures in medical product development emphasizes the need to gather direct patient input as part of early qualitative research to ensure that all relevant concepts are appropriately identified and assessed in future clinical trials [4, 5]. This guidance is supported by the US twenty-first Century Cures Act, which was designed to help accelerate medical product development and bring new innovations and advances to patients who need them faster and more efficiently [6]. The Act highlights that the lived experience of the patient should be incorporated into drug development where possible.

Prior to the conduct of this study, a targeted literature review was undertaken to identify published research studies that qualitatively explored the lived experiences of UC with a focus on the most pertinent patient reported symptoms and HRQoL impacts. Seventeen published qualitative papers were identified as part of the literature review [7–23] which led to the identification of 10 key symptoms and 13 key areas of HRQoL impact relating to UC. The most common patient-reported symptoms were frequent bowel movements (12/17), pain (9/17), sudden/urgent bowel movements (8/17), feeling gassy and passing gas (5/17), and blood in stools (4/17). A number of symptoms were identified in only a few of the reviewed papers, and other symptoms were not consistently reported across the studies, these were: vomiting (3/17), stomach cramping (3/17), incontinence/leaking (3/17), stool consistency (2/17), and mucus in stools (1/17).

Furthermore, eight of the 17 studies included participants with Crohn’s Disease (CD) (or other similar, non-specified, irritable bowel disease [IBD] conditions) as well as participants with UC. In all these studies, the authors did not analyze data according to the specific condition and therefore, it is not possible to differentiate findings attributable to UC versus other IBD conditions. Lastly, only one study explored symptoms associated with adolescent UC [14], however, per the aforementioned studies, the authors did not delineate study findings between adolescents and adults or between UC, CD, or other IBD conditions.

The literature review also revealed the absence of any published conceptual disease model that captured the symptomatic and HRQoL experiences of adults and adolescents living with UC. A conceptual model is important because it can help provide the rationale and definition of the key outcomes of interest, as well as the hypothesized relationship between outcomes, which can then inform decision-making [24]. Thus, a preliminary conceptual model of the key symptoms and HRQoL impacts of UC was developed based on the literature review findings. However, at this stage it was not clear if the symptoms and HRQoL impacts were specific to UC or other IBD conditions due to the mixed populations included in the reviewed studies. Similarly, it was neither clear if any symptoms or HRQoL impacts are attributable to age-specific populations. Subsequently, it was agreed that the newly-developed, preliminary conceptual model required further validation to explore the validity and relevance of the 10 UC symptoms and 13 HRQoL impacts and confirm if any of these are uniquely associated with either adult or adolescent UC.

To achieve this, a qualitative study was initiated involving open-ended interviews with adults and adolescents with UC to 1) explore UC experiences in general and 2) identify any similarities and differences in the symptoms and HRQoL impacts reported by adults and/or adolescents with UC.

## Methods

### Study design and participants

In-depth, face-to-face, semi-structured interviews were conducted with adults and adolescents with clinically-confirmed UC [25–27]. The study design was not restricted to a single qualitative, methodological framework, but was more unstructured to allow for an open exploration of the participant experience of UC.

### Recruitment and sampling

Patients were identified using purposive sampling via clinician referral using a third-party recruitment agency in the US. Clinicians reviewed their site database to identify potential participants that met the eligibility criteria for this study. Participants were eligible if they were fluent in US-English and had UC confirmed by sigmoidoscopy or colonoscopy > 3 months ago. Participants were excluded if they had a previous diagnosis of CD or another form of colitis, had surgical resection that significantly treated their UC, or had a significant or uncontrolled psychiatric or physical comorbid condition that in the opinion of the investigator may interfere with participation in the study. Participants in remission over the last 6 months were also excluded from this study. Adult participants were identified from Chicago, St. Louis, New Orleans, and Baltimore, and adolescent participants were identified from Chicago. Potentially eligible participants were contacted about the study face-to-face and presented with an informed consent form and given the opportunity to ask any questions about the study.

### Interviews

A semi-structured discussion guide, based on the preliminary conceptual model of adult and adolescent UC, was developed. Example questions from the discussion guide are presented in Table 1. The guide was primarily focused around discussion of participants’ symptom experience with UC and its impact on their HRQoL. The questions were developed by a study team of highly experienced qualitative researchers with feedback from a clinician with expertise in UC and an external consultant with expertise in pediatric and adolescent qualitative research. The questions were designed to allow the participants to spontaneously discuss areas relating to the experience of living with UC before being probed on specific symptoms and impacts included in the preliminary conceptual model (if they were not already discussed sufficiently).

**Table 1:** Symptom concepts and supporting quotes

1.	Okay, so you were asked to make a collage to reflect your life with <participant’s term for UC> or how you feel about <participant’s term for UC>. Do you have that with you? Now could you tell me about the images here.
2.	How else would you describe that feeling? What other words would you use to talk about it?
3.	Do you ever have times when your <participant’s term for UC> is worse than usual? Can you walk me through a one of those days, what is it like in the morning/afternoon/evening?
4.	Can you tell me more about how your <participant’s term for UC> affects what you do day-today?
5.	I’m going to ask you to be a bit creative now. Can you think about your <participant’s term forUC> is if it were an animal? What animal best represents your <participant’s term for UC>?Why is that?

Face-to-face interviews were conducted by a highly experienced female interviewer (fourth author) who has substantial experience of qualitative interviewing. The interviews lasted approximately 60 min and were conducted in a conference room of a local hotel, at a time that was convenient to the participant. The interviewer aimed to develop a rapport with the participant prior to the interview commencing to relax the participant and encourage open discussion. Interviews began with open-ended questions designed to elicit spontaneous discussion, which was then followed by further in-depth probing around key areas of interest. Additionally, prior to the interview day, participants were asked to create a collage containing pictures, text or images selected to reflect their personal experience of UC, and to bring this collage with them to the interview to facilitate initial discussion. However, it was explained to participants during the consent procedures that completion of the collage was not a requirement for participation in the study. If a participant brought a collage to the interview, the interviewer asked the participant to explain what each image represented, to allow this information to facilitate later coding. Another creative task used in this study involved asking participants to think about an animal which might represent their UC, and to explain their choice of animal. The value of creative tasks (such as collages and the animal task) to qualitative research has been shown to be particularly useful when interviewing younger participants in order to prompt discussion and orientate the participant to think about key areas of concern relating to their disease ahead of the interview [28]. Given the potential benefits of such a task, it was agreed to extend both tasks to adults as well as adolescent participants.

### Sample size and saturation

Following the initial review of the literature and the authors’ experience of how many interviews are typically needed to reach saturation in homogeneous populations [29], it was anticipated that saturation of concepts would be met when approximately 21 adults (≥18 years) and 19 adolescents, aged 12- to 17-years old had been recruited. However, interviews would continue until saturation was met. Saturation was analyzed for adults and adolescents separately. To determine if saturation was met in this study, participants were divided into three equal sets (three sets of 7 adults; two sets of 5 and one set of 4 adolescents) based on when the participant was interviewed. Saturation was first analyzed for the adult sample; concepts arising in the adolescent sample saturation was evaluated to confirm that the most important symptoms and HRQoL concepts were identified in the interviews and no new concepts would arise with further data collection [30]. Saturation was considered to have been met when no new concepts were discussed in the last set of interviews within each age group [30].

### Analytical approach

All interviews were transcribed verbatim and analyzed using inductive thematic analysis [31]. All coding was conducted by two highly experienced qualitative researchers (second and fourth authors), who have undertaken many qualitative studies and worked as qualitative researchers for many years. All coding was undertaken using the qualitative software ATLAS.ti version 7.0. All data were de-identified before analysis was conducted. There were six steps to thematic analysis that were followed in the current study; although they may appear linear, this is a flexible and reflective process, which, if necessary, during the coding and analysis process steps, could be revisited [31]. The steps followed were:

1. Familiarization – reading and re-reading the data and noting initial ideas;
2. Generating codes – coding interesting features systematically;
3. Identifying themes – collating codes into potential themes;
4. Reviewing themes – checking the themes work in relation to the coding, data, and research objectives;
5. Defining themes – refining the themes to make them specific and clear;
6. Report production – selecting clear and vivid examples that relate back to the research questions in a scholarly report.

Collages were used to help prompt discussion during the interview and to identify the most salient symptoms and impacts associated with UC. Analysis of collage content involved using thematic analysis of participants’ descriptions of the images included in their collage [31]. In addition, the codes generated through analysis of the transcripts were applied to the participants’ collages in order to examine what the participant felt each image represented in relation to their UC.

Finally, for data generated via the animal task, thematic analysis was applied in line with all other qualitative data analysis [31]. The animal task was designed to facilitate spontaneous discussion around participants’ most important experiences of their UC and generate insight into the nature of UC from the patients’ perspective. Thus, the critical part is not the specific animal chosen but the concepts/experiences represented by the selected animal.

All qualitative data were interpreted to understand the experience of participants living with UC in totality, as well as to explore any difference between the two age populations.

The accuracy of thematic analysis [31] was confirmed by comparing the independently identified codes of the two coders on two transcripts for the level of agreement. The two coders also regularly discussed and reviewed each other’s coding through the process to ensure that codes were being developed and applied consistently across the transcripts. In addition, the first author reviewed all of the coding for consistency and suitability. Following finalization of the themes, saturation was evaluated to confirm that the most important symptoms and HRQoL concepts were identified in the interviews and no new concepts would arise with further data collection [30].

### Ethics

All study documents were submitted and approved by the US *Copernicus Group* Independent Review Board®. The study was performed in accordance with the Declaration of Helsinki [32]. Participants and their referring clinician received remuneration for their participation. Prior to study participation, those over 18 years of age were required to review and sign an informed consent form. In comparison, adolescents were asked to review and sign an informed assent form while their parent/guardian was provided with a parental permission form. Consent or adolescent assent and parental permission was collected face-to-face, with all individuals given the opportunity to ask questions and discuss the study. All individuals were reminded that they could withdraw their consent/assent/parental permission at any time before, during, and after the interview.

## Results

### Sample demographics

In total, 21 adults and 14 adolescents were recruited for this interview study, no adults withdrew or dropped out of the study. Among the adult sample, 11 (52%) were female and 14 (67%) were white. Among the adolescent sample, 7 (50%) were female and 10 (71%) were white. Severity of UC over the past 6 months was reported by clinicians as either mild, moderate, or severe. For adults, 5 (24%) were reported as having mild UC severity, 8 (38%) with moderate, and 8 (38%) with severe. For adolescents, 9 (64%) were reported as having mild UC severity, 4 (29%) moderate, and 1 (7%) severe. For adults, the mean number of years since diagnosis was 9.3 (SD = 16.1), and the mean number of hospitalizations due to UC over the past 6 months was 3.0 (SD = 14.3). For adolescents, the mean number of years since diagnosis was 3.0 (SD = 3.1) and the mean number of hospitalization due to UC over the past 6 months was 3 (21.4%).

### Saturation

Saturation analysis was undertaken on all identified concepts for both adults and adolescents separately. Saturation analysis demonstrated that no new themes or descriptions of concepts were introduced within the last group of interviews. Thus, saturation was deemed to have been met for the both adult and adolescent samples and in relation to both symptom and impact concepts. Of note, the symptom ‘rash’ was not included in the saturation analysis as it was excluded from the conceptual model of UC. Following discussion with an expert clinician, they suggested that based on the participants quotes and their understanding, this was likely the result of treatment rather than UC directly. This analysis also highlighted that while saturation of all symptom concepts was reached in both the adult and adolescent samples, two impact concepts (‘impact on school’ and ‘feeling dehydrated’) were only discussed by only adolescents or only adults, respectively.

### How did patients characterize their UC?

At the start of the interview, participants were asked to describe their UC and talk about what it is like to live with UC every day. All but one adult (due to time constraints) (*n* = 20) and all adolescents (*n* = 14) were asked to “*select an animal that best represents your UC*”. This question was purposively open-ended to draw out what might be most important or relevant to participants. The participants were not aware that they would be asked this question, and were free to select any animal they wished, with no proposed response options. Participants were then asked why they selected that particular animal and what it represented. The critical part of this exercise was not the specific animal chosen, but the concepts/experiences represented by the animal that was selected by the participant.

Overall, there were many similarities between the animals chosen by adults and adolescents. Several animals were selected because they reflected the nature or the character of UC. For instance, 6 adults and 4 adolescents selected animals that reflected the ‘unpredictability’ of UC flares or attacks (e.g. bear, koala, snake, octopus, shark). “*A bear. And I’m gonna say a bear because a bear can be cute and cuddly and calm and then it can be extremely aggressive (laughs), um, without being provoked at all*.” NO-001-A-M; 6 adults and 3 adolescents selected animals reflecting the ‘aggressive nature’ or severity of UC (e.g. bear, wolf, gorilla, lion, shark). *“[shark] so kind of like the roar … like it could happen at any time.”* CH-005 A-F; and 2 adults chose animals reflecting the sense of ‘control’ that their UC has over their lives (e.g. lion). “*Lion, King of the Jungle. Controls all beasts*” CH-008 -A-F″.

Other animals were chosen because they represented various symptoms associated with UC, for instance, the frequency of bowel movements/pooping (e.g. monkey, dog, cow) (*n* = 3 adolescents) “*A cow? ... Because they, um, they poop out a lot... and it stinks*” CH-013-a--M; feeling bloated (e.g. hippo, whale) (*n* = 1 adult); blood in stools (e.g. wolverine) (*n* = 1 adult) *“um, they’re very vicious. Um, and just kind of the image of blood comes in just with dealing with this. You get a lot of dealing with blood*.” SL-005-A-M; urgency (e.g. puppy or bunny, rat) (*n* = 1 adult and *n* = 1 adolescent); and pain (e.g. porcupine) (*n* = 1 adolescent) “*Kind of like the pain, you know, like when it does that [mimics fire quills] I get pain*.” CH-012-a-M.

Animals were also chosen because they represented the HRQOL impacts of their UC, such as the emotional aspects of living with UC, for example, feeling irritated (e.g. badger) (*n* = 1 adult). “*Um, because nobody likes them Um, they’re nasty. (laughs) They’re not clean, which is, you know, how I feel when I’m going through one of these attacks*” NO-004-A-M; feeling unclean/dirty (e.g. rat) (*n* = 1 adult); feeling moody (e.g. tiger) (*n* = 1 adult); feeling anxious (e.g. kitten) (*n* = 1 adolescent). “*I would pick my um, 8 month year old pussy because he’s a little anxious and he likes to do the most, and he likes to ... like, I don’t know how to put this, he likes to destroy things*?” CH-015-a-F.

Finally, some participants selected animals because they represent the more distal impacts of UC. One adult selected an animal to represent the lack of sleep due to UC (e.g. horse). “*What’s an animal that just don’t sleep a lot? Is it a horse?*” BM-005-A-M, whereas another adult selected an animal to reflect hiding UC from those around you (e.g. bear) “*Probably a bear. I’m very defensive about it. I’m very protective. I don’t want my, you know, my little kids don’t know about it*” NO-005-A-F. One adolescent selected an animal to reflect feeling socially isolated (e.g. Tasmanian devil) “*Um, like, I’ve heard, like, there’s not many Australian daredevils, like, they’re kind of going extinct... and like, I haven’t- I’ve never really met anybody else with my stomach condition*” CH-011-a-F.

**Fig. 1:**
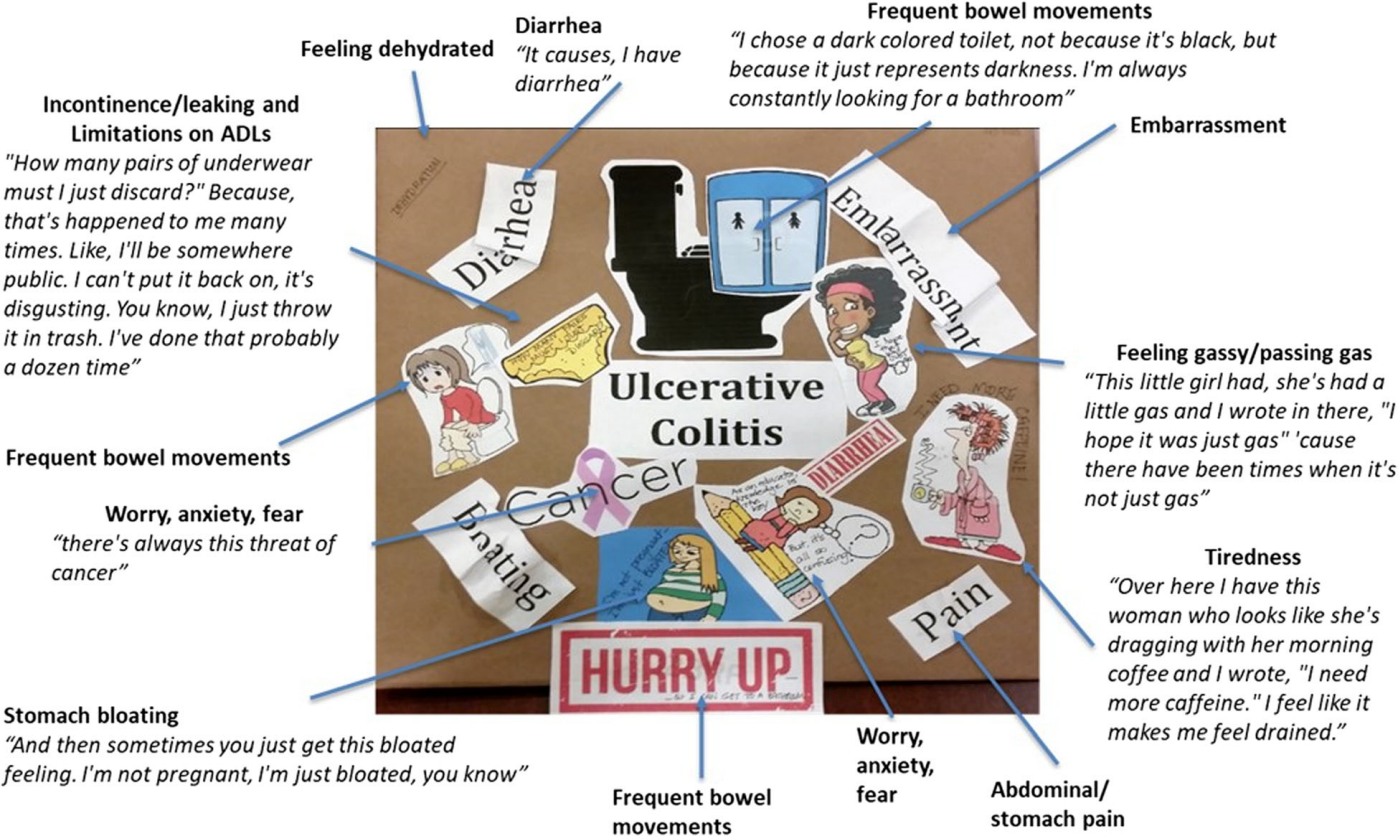
Example of a participant’s collage from this study

Prior to the interview, participants were asked to bring a collage with them to aid interview discussion. Although this was optional, 31 participants (89%) completed this task (17 adults (81%) and 14 adolescents (100%) brought a collage to their interview. The language participants used to describe images in the collages was used to aid coding. The collages helped identify the most salient symptoms and impacts associated with UC and supported the findings from the interview discussion. The images included in the collages provided valuable insight into the experience of participants living with UC (Fig. 1). For example, pictures of toilets and/or toilet stalls were used to depict the frequency of bowel movements, pictures of characters running to the restroom were used to reflect the urgency of having a bowel movement, and pictures of characters yawning or looking sleepy were often used to reflect fatigue. To capture pain, most participants tended to write the word “pain” across their collage. There were no clear differences between adults and adolescents in terms of the content of the collages.

### Identified themes and development of a patient-centered conceptual model of UC

**Fig. 2:**
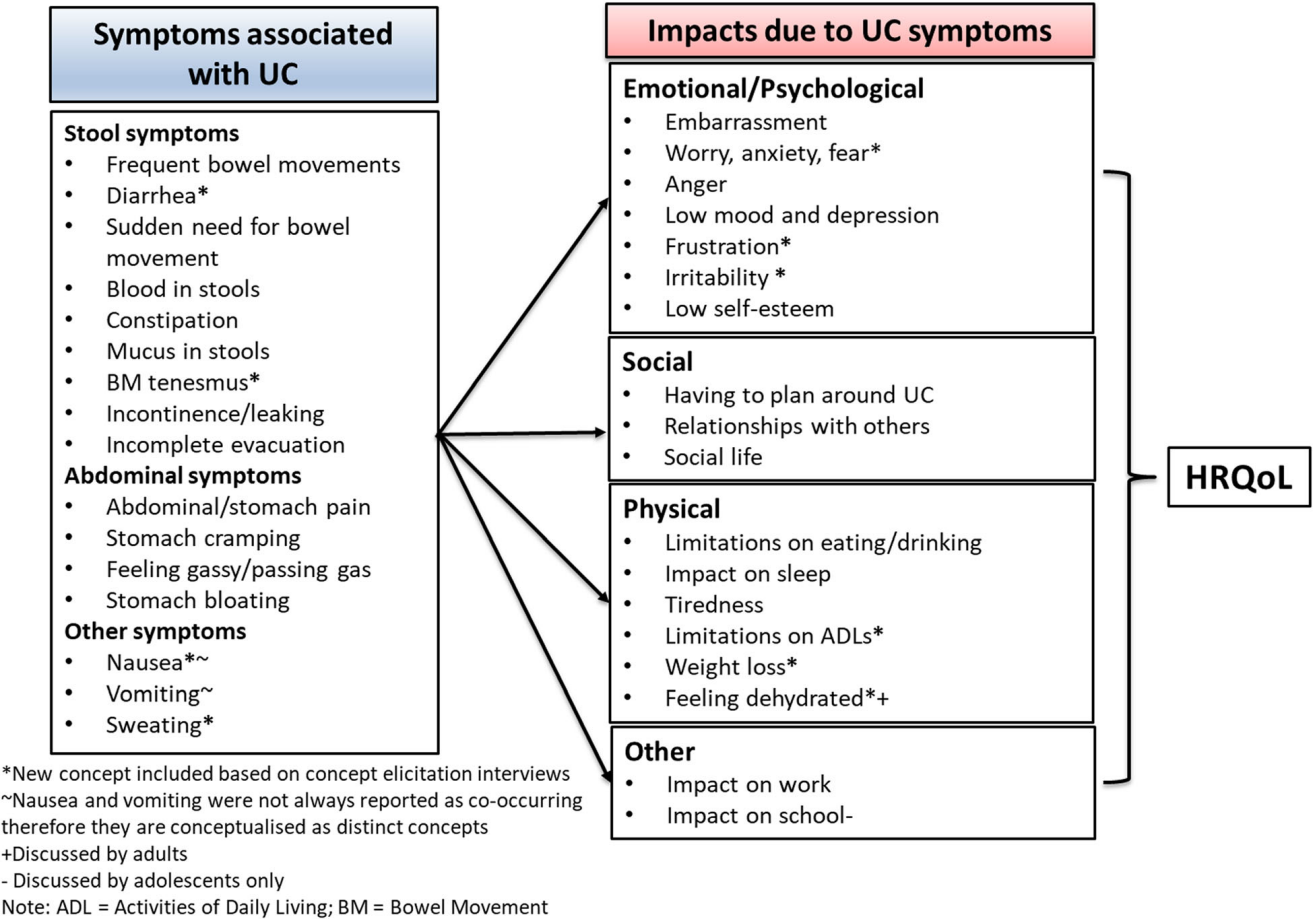
Revised conceptual model of adult and adolescent UC symptoms and HRQoL impacts

Based on the participants’ responses to the animal task, collage task, and a full qualitative analysis of the interview data, a substantial burden associated with UC for both adults and adolescents was revealed. Several themes relating to symptoms and impacts were identified as important based on participants’ descriptions of their experience of UC. Figure 2 shows the revised conceptual model, including the most important and relevant UC symptoms and impacts that were identified by participants during the interviews.

Tables 2 and 3 present information on all the themes relating to the symptoms and HRQoL impacts identified, respectively, along with a selection of supporting quotes from both adults and adolescents.

**Table 2:** Symptom concepts and supporting quotes

Concept included in revised conceptual model of UC	%	Example quote from adult participant	%	Example quote from adolescent participant
Abdominal/stomach pain	100%	“*Yeah. Uh, m-most of my pain will be in the abdomen and the lower quadrant”* CH-003-A-M	100%	“*... It’s kind of like a sharp, sharp pain. ..and just ... It’s pain … right here on my abdomen ... That area.”* CH-012-a-M
Stomach cramping	*81%*	*“an extreme amount of abdominal cramping. I can’t lay on either one of my sides, the left or the right side*.” BM-002-A-F	93%	“*no one wants that cramp, tightening, balled up, like somebody’s stabbing you in your stomach feeling*.” CH-002-a-F
Stomach bloating	86%	“*Yeah, it tends to get harder and kind of swollen in that area [points to stomach]. Um, and if my flare continues it kinda turns into I’m just bloated all day long and it’s kind of uncomfortable to eat ‘cause the food doesn’t hurt me, but it just, I guess filling up my stomach on top of the bloatness is just uncomfortable.”* NO-001-A-M	78%	“*Uh, usually it’s like when you feel bloated, like you’ve just- … like ... Like when you’re, like, extremely full*” CH-011-a-F
Frequent bowel movements	90%	“*And I was going to the bathroom [for a bowel movement], like, every hour all day*.” BM-001-A-F	93%	“*in my physics class I used the bathroom [for a bowel movement] three times and my class was 45 min*.” CH-010-a-F
Sudden need for bowel movement	90%	“*That’s the urgency is because your muscles can’t hold tight enough to prevent the liquid [diarrhea] from coming out … I’ve got to go now because it’s just gonna come out*.” SL-002-A-F	78%	“*And then I just feel like, I don’t know, like I feel like I just get a 10 s timer and once I feel it I’m like I need to use the bathroom [for a bowel movement]*” CH-015-a-F
Incomplete evacuation	*38%*	*“And then once it comes out [bowel movement], you’re like, “Okay, I think it’s done.” You’re like, “Nope. Not done. I’m going to sit here for another 10, 15 min.*” SL-002-A-F	43%	“*it feels like you need to stay on there but you really don’t need to*.*.*” CH-009-a-M
*Bowel movement* tenesmus	38%	“*I’ll have to feel like I have to go [for a bowel movement], go to the bathroom but nothing*.” SL-005-A-M	64%	“*Sometimes I’ll go to the toilet and it doesn’t come out*.” CH-010-a-F
Incontinence/leaking	19%	^“^*After having the [bowel movement] accident on myself and was just humiliated”* BM-002-A-F	*78%*	*“I was in the middle of the day at school. I had stained my pants really bad*.” CH-010-a-F
Diarrhea	86%	“*It’s like pure ... like just fluid water [during a flare]*” SL-002-A-F	100%	“*Uh, I want to say like- like diarrhea, but like I don’t really know how to explain it*” CH-016-a-M
Constipation	71%	^“^*So, I just kind of be clenching for it all day, just waiting to see, am I going to be constipated, am I going to have diarrhea*?” BM-001	78%	^“^*It kinda gets me constipated sometimes*.” CH-001-a-M
Feeling gassy/passing gas	86%	“*... sometimes I would lay on my side then to try and pass some of the gas … gassy and feeling all hurt all over my sides ‘cause it’s just too much air inside of my body .*..” BM-002-A-F	78%	“*Gas, yeah. I get gassy sometimes*.” CH-004-a-M
Mucus in stools	*67%*	*“Sometimes it was just, like, the blood and the mucus that was just coming out*.” NO-006-A-F	*21%*	^*“*^*Um, like mucus, blood sometimes ...*” CH-006-a-F
Blood in stools	90%	^“^*Yeah, typically the biggest symptom is blood in my stool*.” SL-005-A-M	78%	“*… when it’s like semi-solid … usually there’s blood*.” CH-004-a-M
Nausea	33%	^“^*I’ll actually start to feel a little nauseated*” NO-004-A-F	7%	“*Participant: it’s really hard for me to eat [because of my UC]- Like- like I- I think I’ve tried it [food] before and like I think- I think I puked it up* *Interviewer: Oh, okay. So it made you nauseous?* *Participant: Huh? Yeah, it just like did not feel good [nauseous after eating foods]*.” CH-016-a-M
Vomiting	*29%*	*“The pain gets to the point where it’s so bad that it forces me to throw up*.” NO-002-A-M	21%	“S*ometimes, on some cases, I, like, throw up*.” CH-011-a-F
Sweating	19%	“*I can like start sweating in the middle of the class in front of the kids*.” NO-001-A-M	7%	“*Uh, It kind of just feels like you’re sweating … really sweaty and stuff*.” CH-011-a-F

*UC* ulcerative colitis, *a* adolescent, *A* adult, *F* female, *M* Male

**Table 3:** Impact concepts and supporting quotes

Concept included in revised conceptual model of UC	%	Example quote from adult participant	%	Example quote from adolescent participant
Emotional/Psychological Impact				
Low mood and depression	76%	“*I’m so drained, I’m so down, I’m so dealing with this thing, it’s so all-consuming, you know*” NO-005-A-F	78%	“*… like I feel sad sometimes about having a condition*” CH-002-a-F
Worry, anxiety, fear	81%	“*But yeah, I worry all the time.”* BM-005-A-M *“And the reason I feel scared is because I never know if I’m going to have to get up in the middle of something and-it’s.. Just living with complete uncertainty*” CH-008-A-M	86%	“*is a, [picture of] a girl that looks like really scared, or like worried about th- something. And so I picked that, because like I honestly never know when it [UC attack] could happen, so it’s like, kinda like worries me*” CH-008-a-F
Embarrassment	*90%*	*“Embarrassment sometimes because you don’t really know, you know, how to describe it to people*” SL-003-A-F	*86%*	*“It just makes me embarrassed*.” CH-009-a-M
Low self-esteem	24%	^“^*I just feel, like, gross, kinda (laughs). Like my, just, my stomach doesn’t feel good, and, and, like, I don’t feel sexy or (laughs) anything like that.”* NO-006-A-F	36%	“*Just like kinda like, always like worrying about like, what people think, and like it’s kinda like embarrassing, so like I kinda wanna like keep it inside*.” CH-006-a-F
Anger	52%	“*But I was angry in the beginning. I would say why me*?” CH-008-A-M	57%	“*Makes me angry that I have to miss out on so much learning I could be using*” CH-001-a-M
Frustration	*52%*	*“Because you can’t leave your house, or you can’t go out, and that’s extremely frustrating*.” NO-002-A-M	43%	“***…*** *it is kind of frustrating … ..Like that I can’t really do … there’s really no like real treatment*.” CH-004a-M
Irritability	43%	“*Because it made me very anxious and irritated*” BM-002-A-F	24%	“*I just be like, “Really?” Like, like I be crying sometimes, because I just be like, “Oh, my God. This is so irritating.” Like, I don’t want to go through but I have to*.” CH-002-a-F
Social Impacts				
Social life	38%	“*For me, it, it uh, it’s really difficult to, to have to cancel plans and, and not go out to things, and not see the friends that I want to see. Um, because of a, a flare up.”* SL-005-A-M	78%	“*Um, maybe when my friends want to go out and I’m just ... I just say it like I don’t feel well because otherwise if I do go out and I have those pains I would kind of be embarrassed”* CH-012-a-M
Relationships with others	71%	“*Now, with my family, they, th*-*they, they’ve been through this for a while so they kind of understand that there’s ... there’s that unpredictable, unpredictability that can happen. But uh, basically if it [UC] happens I’m going to stay home. I’m going to end any event ... any things that I was going out to a dinner, to a movie or show ... No. You can’t do that*” CH-003-A-M	93%	“*I don’t really try going out and doing anything like my family wants to go out to dinner like I’ll kind of just stay home.”* CH-014-a-M
Having to plan around UC	85%	“*I’m afraid if I go anywhere is that I’ll have to find a bathroom … I don’t like to go to anyone’s house because I’m so embarrassed to go to anyone’s bathroom.”* SL-003-A-F	78%	“*I mean, my parent’s probably try to like plan around it, but ... there’s not much you can do*.” CH-006-a-F
Physical impacts				
Limitations on eating/drinking	90%	“*Like sometimes you can have pain, like I can have some pain here. But if you add alcohol to that pain, then it intensifies the pain*” BM-003-A-F	71%	“*So like by controlling what I eat like a lot of stuff is like it makes a lot easier to deal with*.” CH-004-a-M
Impact on work	85%	“*One time I was at work and all of the sudden it came on me just real fast and I got it all over the walls, all over*” NO-003-A-F	28%	“*it was hard at first and they’re like, really? Are you going to keep calling in? How often are you going to call in? I get really stressed, I’m like, I don’t want to call in to work right now...”* CH-018-a-F
Limitations on ADLs	24%	^“^*It will impact my daily routine if I want to go out and do recreational activities, it will impact that*” CH-008-A-M	57%	“*I feel my stomach hurts, I can’t do anything”* CH-017-a-F
Tiredness	43%	“*Um, and the tiredness, it just wipes you out. I can honestly say, I can remember early in the beginning of it all, I didn’t know where the tiredness came from, you know*” BM-002-A-F	24%	“*Um, it [UC] gets me pretty- pretty exhausted, um, and I’m like tired*..” CH-016-a-F
Weight loss	*19%*	*“I mean, with losing the weight, I get*” SL-005-A-M	43%	“*Over the course of like me having this disease, I’ve lost a lot of weight*.” CH-016-a-M
Feeling dehydrated	*19%*	*“I feel usually dehydrated [just before a UC flair] so I can tell something’s up.”* CH-007-A-M	N/A	N/A
Other Impacts				
Impact on school	N/A	N/A	86%	“*That day was really bad. I ended up leaving school early. I was in the middle of the day at school. I had stained my pants really bad*.” CH-010-a-F
Impact on sleep	62%	^“^*Yeah, tough to fall asleep, and then, like, if I move and I wake up, like, last night I woke up to go to the bathroom, and then I just ... I couldn’t fall back asleep*” NO-006-A-F	93%	^“^*Yeah. It wakes me up in the middle of the night and I’m like, no. Now I can’t sleep*.” CH-018-a-F

*ADLs* activities of daily living, *N/A* not applicable, *UC* ulcerative colitis *a* adolescent, *A* adult, *M* Male, *F* Female

The only symptom spontaneously reported by all 21 adults and all 14 adolescents was stomach/abdominal pain. This was described by participants in both groups as “cramping pain”, “stabbing pain”, “sharp pain”, and “burning pain”. It is important to note that “abdominal/ stomach pain” and “cramping” are conceptualized as distinct symptoms because participants described stomach cramping as always being painful, but not all stomach/abdominal pain involved a cramping sensation. Most participants used the terms “abdominal” and/or “stomach” to describe where the pain occurs. In both groups, “stomach” was more frequently used (*n* = 18 adults and *n* = 14 adolescents) than the term “abdominal” albeit that often the terms were used interchangeably when discussing the symptom.

Other symptoms that were reported by ≥75% of all participants included frequent bowel movements (*n* = 19 adults and *n* = 13 adolescents), diarrhea (*n* = 18 adults and *n* = 14 adolescents), blood in stools (*n* = 19 adults and *n* = 11 adolescents), sudden need for a bowel movement (*n* = 19 adults and *n* = 11 adolescents), stomach cramping (*n* = 17 adults and *n* = 13 adolescents), bloating (*n* = 18 adults and *n* = 11 adolescents), and feeling gassy/passing gas (*n* = 18 adults and *n* = 11 adolescents). In contrast, the symptoms that were discussed least frequently were vomiting (*n* = 6 adults and *n* = 3 adolescents), nausea (*n* = 7 adults and *n* = 1 adolescent), sweating (*n* = 4 adults and *n* = 1 adolescent), and rash (*n* = 2 adolescents).

Most participants (adults and adolescents) reported experiencing symptoms on a regular basis, however some symptoms were reported as occurring only during acute attacks or UC “flares”. The symptoms discussed by adults and adolescents as occurring during a flare only were stomach cramping, blood in stools, mucus in stools, vomiting, and sweating. In contrast to this, all other symptoms were discussed as daily occurrences.

The ways in which participants talked about their UC experiences were similar for both adults and adolescents, with the exception of two symptoms: feeling bloated and incontinence/leaking. Adolescents did not tend to describe how long they felt bloated which contrasted with adults who described feeling bloated for “*a couple of hours*” to “*2 months*” (see Table 2 for example quotes). Adults described incontinence/leaking as a regular symptom, with two adults discussing that they use adult diapers and incontinence pads to address this symptom. “*But I have had accidents, so that’s very, very, very frustrating. And also just the whole having to even wear a diaper sometimes is very frustrating*” NO-002-A-M. In comparison, adolescents described ‘incontinence/leaking’ as an infrequent symptom and did not discuss the use of diapers and incontinence pads, albeit it was not probed by the interviewer specifically. “*So, if I wake up on time, I’ll just use the bathroom and try to go back to sleep. But if I don’t then I’ll like stay up and I’ll like wash my underwear and stuff and then I’ll like take a quick shower and like just feel like, try to feel clean*” CH-015-a-M.

When reviewing the HRQoL impacts discussed by participants, no impact was discussed by all participants. However, ≥75% of participants reported feeling embarrassed because of their UC (*n* = 19 adults and *n* = 12 adolescents), having to limit their diet (*n* = 19 adults and *n* = 10 adolescents), having to plan around UC (*n* = 18 adults and *n* = 11 adolescents), feeling worried, scared, or angry about UC (*n* = 17 adults and *n* = 12 adolescents), low mood and depression (*n* = 16 adults and *n* = 11 adolescents), as well as UC having a negative impact on their relationships with others (*n* = 15 adults and *n* = 13 adolescents). All impacts were discussed by both adults and adolescents except for ‘feeling dehydrated’ (mentioned by 4 adults only) and the ‘impact of school’ (mentioned by 12 adolescents only). The HRQoL impacts discussed by participants can broadly be grouped into three categories: emotional/psychological impacts; social impacts; physical impacts; and others, see Fig. 2.

The ways in which participants talked about the HRQoL impacts relating to their UC were similar for both adults and adolescents with the exception of two impacts: impact on school and dehydration. Only adolescents discussed that their UC had an impact on school “*you go into the bathroom [when at school and] it’s kind of like you’re stuck there. And you don’t really know if you’re going to be able to come out in five, ten minutes or you’re going to be sitting in there for 20, 25, 30 minutes*.” CH-014-a-M. No adult participants reported being in school/college at the time of the interview, which is likely the reason why this concept was not discussed by adult participants. The concept of feeling dehydrated was only discussed by adults “there’s a dehydration part that can happen” CH-003-A-M. It is not clear why adolescents did not report this as it was not probed further during the interviews. Interestingly, both adults and adolescents discussed ‘impact on work’, with some adolescents reporting being in some form of employment which was impacted by their UC.

When discussing HRQoL impacts the occurrence of flares or acute attacks were described as being particularly burdensome; participants reported that they try to avoid triggering these attacks as much as possible by adapting what they eat and drink. *“It’s what kind of foods you kinda eat [triggers attacks]”* CH-010-a-F. When an attack did occur, participants talked about avoiding going out at all and staying at home to deal with their UC symptoms. Staying at home meant that they were more comfortable, closer to a toilet, and not in a situation where they felt like they might be judged by others. *“Um, and it’s just certain times. I, I stayed in the house 1 month at a time when it was really out of control, not able to leave the house.”* BM-002-A-F.

The conceptual model that was initially developed from the literature was updated to reflect findings from analysis of interview data to include four additional symptoms that had not been identified from the literature. These were: ‘bowel movement tenesmus’, ‘diarrhea’, ‘nausea’, and ‘sweating’. All other concepts in the initially developed conceptual model were also identified in the qualitative data. No participants used the term ‘tenesmus’, rather it was described as a feeling of needing to have a bowel movement but no bowel movement would pass; since this is clinically referred to as tenesmus, the term was used in order to be concise within the conceptual model.

The interviews also led to a more detailed understanding of specific concepts of pain (abdominal/stomach pain, cramping pain) and stool consistency (constipation and diarrhea). These concepts were separate in the updated conceptual model. The concept ‘rash’ was not included in the conceptual model because it was not frequently reported, and for the two adolescents who reported having a skin rash, both described it as occurring after other symptoms during a UC flare. Following discussion with the expert clinician, they suggested that this symptom was therefore likely a result of treatment rather than UC directly.

Furthermore, five HRQoL impact concepts were added to the conceptual model including ‘frustration’, ‘irritation’, ‘weight loss’, ‘limitations on activities of daily living’, and ‘feeling dehydrated’. Three impacts were refined to better reflect how they were described during the interviews; ‘eating restrictions’ was renamed to ‘limitations on eating/drinking’ to capture the experience of adults who described alcohol restrictions specifically, as well as food and diet limitations; the term ‘fear’ was added to the concept of ‘anxiety/worry’ as fear was described as an extreme form of worry; and lastly, ‘fatigue’ was renamed ‘tiredness’ as the latter term was more typically used by participants in the interviews.

## Discussion

Overall, analysis of qualitative data from the interviews revealed a substantial physical and emotional burden of UC as well as its impact on participants’ lives. It is clear from this study that UC has a profound impact on adult and adolescent lives, with many of them discussing the severe nature of UC which can be both unpredictable and controlling. This study has allowed for the refinement of the initially developed conceptual model, which highlights the 16 UC symptoms that were most frequently reported by participants, as well as the 18 HRQoL-related concepts. Almost all concepts within the model were mentioned by both adults and adolescents, indicating that there is a strong overlap in the adult and adolescent patient experience of UC. Thus, the experience of UC may not be dependent on age, but rather symptoms and experiences may be similar regardless of age. This was also reflected in the animal task whereby both adults and adolescents selected animals to depict how they experience UC in terms of symptom and HRQoL impacts.

While many of these symptoms were described as occurring on a regular basis, some were only experienced during acute attacks or UC “flares”, such as stomach cramping and vomiting. This is important to understand to ensure that the measurement strategy considers the frequency or timing of certain symptoms. A few symptoms, such as vomiting and sweating, were more common among adults suggesting they are more likely to be associated with UC in adulthood. It could also reflect that the adolescents in this study had less severe UC, as recorded by their clinician, than their adult counterparts. Therefore, these symptoms may be indicative of a more severe condition type, rather than being associated with the age of the patient. In addition, while ‘incontinence’ was reported by both adults and adolescents, only adults described having to wear diapers to prevent accidents. Adults also discussed incontinence as occurring more frequently than adolescents. Again, this could reflect the severity of the symptom and perhaps indicated that the adolescent participants had slightly lower UC severity than the adult participants. It may also be that adolescents were self-conscious or embarrassed to discuss, or even consider wearing protection because of incontinence.

Previous qualitative studies in this area have primarily focused on adult populations, with the majority including participants with CD (or other similar, non-specified, IBD conditions) as well as participants with UC. The inclusion of several conditions makes it difficult to differentiate between conditions and ultimately identify those symptoms that are unique to UC [7–22]. The current study built on these previous studies by interviewing both adults and adolescents. The symptoms and impacts experienced bn adults and adolescents were similar. In addition, the current study interviewed participants with only UC, rather than including those with CD or other IBD related conditions, which has allowed for the development of a disease-specific conceptual model of the patient experience of UC.

The initial conceptual model was developed from previous research which had used participants reporting with UC as well as other conditions such as CD and IBD. Following the current qualitative study, the initially developed conceptual model was updated to reflect the experiences of adults and adolescents with UC. Four symptoms and five areas of impact were identified in this qualitative study that were not identified in the initial literature review. Overall, there was a clear overlap in the adult and adolescent patient experience of UC, which is important when designing future UC clinical trials, particularly those that include different age populations.

The animals task used during the interviews was both helpful and insightful due to it further supporting the way both adolescents and adults experience UC is similar in nature. This along with the use of the collage task helped with discussions particularly in the adolescent cohort. Whilst the animal task also allowed for the identification of the most important factors of living with UC.

### Limitations

Although saturation of concepts was met based on the qualitative analysis performed, the number of adolescents initially targeted for recruitment into this study was not achieved. While 19 were targeted, 14 adolescents were included in the study. Based on clinician feedback, it was likely due to adolescents receiving treatment and reaching remission very quickly, therefore there was less availability of adolescent subjects with ‘active disease’. It is possible that the inclusion of additional adolescent participants could have led to the identification of new or other concepts. However, we feel this is of minimal risk since the conceptual model was first developed from a review of existing literature and then updated based on 35 patient interviews. Additional cognitive interviews are planned to explore the suitability of outcome measures and confirm the validity of the revised conceptual model.

Secondly, adolescents were only recruited from one location in the US, which may impact the generalizability of the findings. For adults, a representative sample was recruited taking into consideration race, ethnicity, and gender. However, adolescent participants had a range of UC severity (although they were less severe than our adult sample) which supports the clinical representativeness of the sample. There is no clinical reason to suggest that more geographic diversity would have led to different concepts being identified.

## Conclusions

Qualitative analysis of the interviews confirmed a substantial burden of UC on both adults and adolescents and identified several symptoms experienced in this population. The similarities in the symptoms and HRQoL impacts discussed by adults and adolescents with UC highlights the comparable nature of the condition in these two age populations, which has greatly added to our understanding of the HRQoL and symptomatic experience of this condition across different age groups.

The newly proposed conceptual model of UC generated as part of this research reflects the experiences of this condition from the perspective of adult and adolescent patients. The next step is to use the newly-developed disease-specific conceptual model and patient experience data to inform drug development and regulatory decision-making for this condition and further validate the conceptual model. The current research has highlighted a clear need for this considering the profound impact that UC has upon the lives of adults and adolescents.

## Data Availability

The datasets generated and/or analysed during the current study are not publicly available due to the sensitive nature of the questions asked in this study but are available from the corresponding author on reasonable request.

## Abbreviations

CD: Crohn’s disease
HRQoL: Health-related quality of life
IBD: Irritable bowel disease
PRO: Patient-reported outcome
UC: Ulcerative colitis
US: United States
